# Admission creatinine level predicts postoperative acute hepatic dysfunction in patients aged ≤30 years with acute type A aortic dissection: a multi-center retrospective study

**DOI:** 10.3389/fcvm.2026.1898082

**Published:** 2026-08-17

**Authors:** Yu Xu, Ling-Jin Huang, Yu-Hao Xia, Zi-Ming Bai, Chang-Bo Xiao, Hong-Qiao Lu, Xiao-Fu Dai

**Affiliations:** 1 Department of Cardiovascular Surgery, Fujian Medical University Union Hospital, Fuzhou, China; 2 Key Laboratory of Cardio-Thoracic Surgery (FujianMedical University), Fujian Province University, Fuzhou, China; 3 Department of Cardiovascular Surgery, Xiangya Hospital, Central South University, Changsha, China; 4 Department of Cardiovascular Surgery, Chest Hospital of Zhengzhou University, Zhengzhou, China; 5 The Affiliated Hospital of Kunming University of Science and Technology, Kunming, China

**Keywords:** acute hepatic dysfunction, acute type a aortic dissection, creatinine (Cr), risk factor, young adult

## Abstract

**Background:**

This study aimed to investigate the risk factors for postoperative acute hepatic dysfunction in patients aged ≤30 years with acute type A aortic dissection (ATAAD).

**Methods:**

This retrospective study included clinical data from patients who underwent surgery for acute type A aortic dissection (ATAAD) at Fujian Medical University Union Hospital, Xiangya Hospital of Central South University, and Henan Provincial Chest Hospital. Eligible patients were divided into an acute hepatic dysfunction (AHD) group and a non-AHD group according to their MELD scores. Univariate and multivariate logistic regression analyses were conducted to identify independent risk factors for postoperative AHD. Subsequently, a receiver operating characteristic (ROC) curve was generated to assess the predictive performance of the identified factors.

**Results:**

A total of 122 patients were enrolled, comprising 100 males (81.97%) and 22 females (18.03%), with a median age of 28.00 years (IQR: 25.00–29.00). Postoperative AHD occurred in 34 patients (27.87%), who were assigned to the AHD group, while the remaining 88 patients (72.13%) formed the non-AHD group. Baseline comparisons revealed significant differences between the two groups in history of hypertension, white blood cell count, neutrophil count, lymphocyte count, lymphocyte-to-monocyte ratio (LMR), pan-immune-inflammation value (PIV), systemic inflammation response index (SIRI), total protein (TP), albumin, creatinine (Cr), prothrombin time (PT), and INR (all *P* < 0.05). Multivariate logistic regression identified Cr as an independent risk factor for postoperative AHD in ATAAD patients aged ≤30 years (OR = 1.04, 95% CI: 1.01–1.06, *P* = 0.002). The ROC curve demonstrated that Cr had good predictive value (AUC = 0.816, 95% CI: 0.727–0.906; *P* < 0.0001), with a sensitivity of 0.765 and a specificity of 0.773.

**Conclusion:**

Elevated admission serum creatinine level is associated with an increased risk of postoperative acute hepatic dysfunction in patients aged 30 years or younger with acute type A aortic dissection. Accordingly, routine assessment and serial monitoring of this biomarker are essential for the early identification of high-risk individuals and for optimizing perioperative management.

## 1 Introduction

Acute type A aortic dissection (ATAAD) is a life-threatening cardiovascular emergency. It is associated with a 1% increase in mortality per hour after symptom onset (1), and the mortality rate can be as high as 55% within 48 h (2). its development is mainly linked to hypertension, smoking, and connective tissue diseases (3). Emergency surgery remains the only effective treatment (4). Although advances in surgical techniques and perioperative life-support systems have reduced postoperative complications, acute liver dysfunction persists as a serious and often overlooked clinical concern (5).

ATAAD predominantly affects middle-aged and older adults and is uncommon in patients aged 30 years or younger. Data from the International Registry of Aortic Dissection indicate that this age group accounts for approximately 1.74%–2.46% of all ATAAD cases (6). Consistently, a large international multicenter study identified only 139 patients aged ≤30 years among 7,914 surgically treated ATAAD patients (1.8%) (7). In these exceptionally young patients, ATAAD is more frequently associated with connective tissue disorders, particularly Marfan and Loeys-Dietz syndromes, although hypertension remains an important predisposing factor in those without connective tissue disease. Notably, Marfan syndrome was present in 44 of the 139 young patients (31.7%) in the aforementioned multicenter study (6–8). Although Marfan syndrome affects both sexes, male patients may have a higher overall risk of aortic events, whereas pregnancy and the early postpartum period confer additional risk in women; Marfan-associated ATAAD is also more frequently accompanied by aortic regurgitation and supra-aortic vessel involvement, without an apparent increase in adjusted early postoperative mortality (9, 10). Compared with older patients, younger individuals frequently present with structural abnormalities of the vascular wall and extensive aortic involvement, often necessitating complex root and arch reconstruction and prolonged hypoperfusion and cardiopulmonary bypass times (11). Meanwhile, the dissection itself can provoke a marked systemic inflammatory response and ischemia–reperfusion injury (12). Coupled with substantial postoperative hemodynamic fluctuations, the liver-being highly sensitive to ischemia and hypoxia—is particularly vulnerable to early postoperative injury (13).

Therefore, research focused on early identification and intervention strategies for postoperative acute hepatic dysfunction in ATAAD patients aged 30 years or younger is of considerable clinical importance for reducing postoperative mortality and improving long-term outcomes.

## 2 Materials and methods

### 2.1 Participants

**Figure 1:**
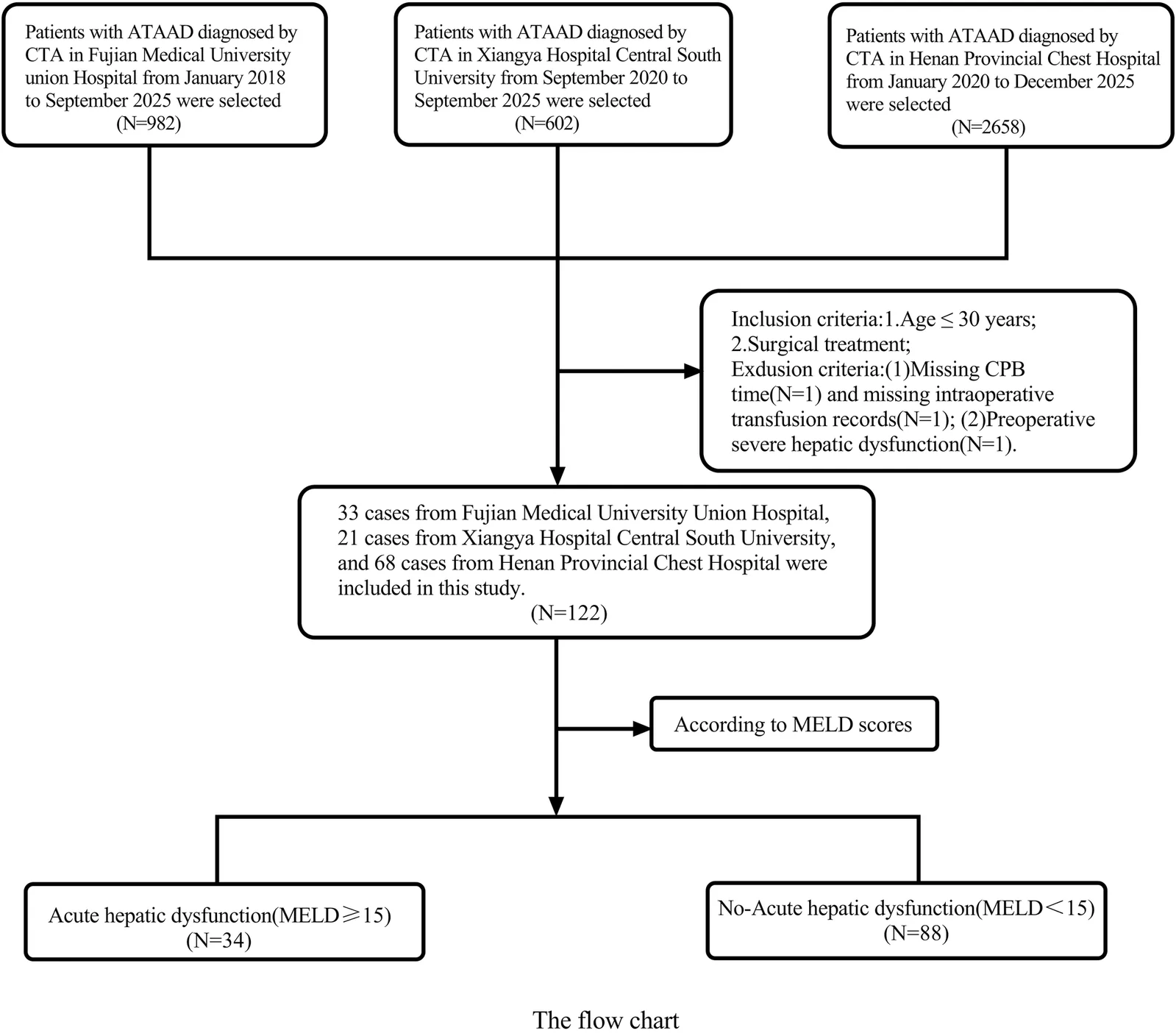
The flow chart.

This study consecutively enrolled ATAAD patients confirmed by computed tomography angiography (CTA) from three medical centers: Fujian Medical University Union Hospital (January 2018–September 2025, *n* = 982), Xiangya Hospital of Central South University (September 2020–September 2025, *n* = 602), and Henan Provincial Chest Hospital (January 2020–December 2025, *n* = 2,658). Patients were included if they met the following criteria: (1) age ≤30 years; and (2) underwent surgical treatment. Exclusion criteria were: (1) missing cardiopulmonary bypass (CPB) time (*n* = 1) or missing intraoperative transfusion records (*n* = 1); and (2) preoperative severe hepatic dysfunction (*n* = 1). Ultimately, 122 patients were included in the final analysis (Figure 1).

### 2.2 Outcome definition

Postoperative AHD is defined according to the MELD score. The Model for End-Stage Liver Disease (MELD) score for liver function was calculated using the formula: 11.2 × ln(INR) + 3.8 × ln(bilirubin) + 9.6 × ln(creatinine) + 6.4 × etiology (14). Since none of the patients had pre-existing liver disease, the etiology term was set to 0 for all cases. Postoperative MELD scores were calculated using the highest values of serum bilirubin, INR, and creatinine measured within the first 72 h after surgery (postoperative days 1, 2, and 3). Notably, the creatinine component in this postoperative MELD score was derived from the highest postoperative serum creatinine value measured within the 72-hour window, rather than the admission creatinine value. For each patient, the three component values corresponding to the highest MELD score within this 72-hour window were used for analysis. Patients were then divided into two groups: hepatic dysfunction (MELD score ≥ 15) and non-dysfunction (MELD score < 5). All patients had complete measurements of bilirubin, INR, and creatinine on at least one time point within postoperative days 1–3, and no patient was excluded due to missing data. In contrast, admission serum creatinine was used solely as a potential predictor (exposure variable) in the subsequent risk factor analysis.

### 2.3 Data collection

Data were collected through the hospital's electronic medical record system, including: Demographic characteristics: gender, age, height, weight, body mass index (BMI), smoking history, drinking history. Past medical history: Hypertension (HP), Diabetes Mellitus (DM), Marfan syndrome. Initial in-hospital indicators: Systolic Blood Pressure (SBP), Diastolic Blood Pressure (DBP), Heart Rate (HR), White Blood Cell count (WBC), neutrophil count, lymphocyte count, monocyte count, Red Blood Cell count (RBC), Hemoglobin (HGB), Platelet Count (PLT), Lymphocyte-Monocyte Ratio (LMR), Pan-immune Inflammation Value(PIV), Systemic Immune-inflammation Index (SII), Systemic Inflammatory Response Index (SIRI), Platelet-to-Lymphocyte Ratio (PLR), Alanine Aminotransferase (ALT), Aspartate Aminotransferase, Total Bilirubin (TBil), Direct Bilirubin (DBil), Indirect Bilirubin (IBil), Total Protein (TP), Albumin, Creatinine(Cr), Activated Partial Thromboplastin Time (APTT), Prothrombin Time (PT), International Normalized Ratio (INR), Fibrinogen (FIB), Thrombin Time (TT), Left Ventricular Ejection Fraction (LVEF). Intraoperative indicators: red blood cell transfusion volume, plasma transfusion volume, platelet transfusion volume, Cardiopulmonary Bypass Time (CPB), aortic cross-clamping time, deep hypothermic circulatory arrest time (DCCA), surgery time. Postoperative indicators: Cr, INR, TBil. Composite index: LMR = lymphocyte/monocyte, PIV = (Neutrophil*Platelet*monocyte)/lymphocyte, SII = (Platelet*Neutrophil)/Lymphocyte, SIRI = (Neutrophil*monocyte)/lymphocyte, PLR = Platelets/lymphocyte. All patients had complete measurements for the component variables of the composite indices (neutrophil count, lymphocyte count, monocyte count, and platelet count). Consequently, no patient was excluded due to missing data for LMR, PIV, SII, SIRI, or PLR.

### 2.4 Statistical analysis

Statistical analyses were conducted with R software, version 4.3.0. The glm function from the base stats package was used to fit the logistic regression models, with the “family parameter set to binomial(link = “logit”). The tableone package was used to generate baseline characteristics tables. Normally distributed continuous variables were expressed as mean ± standard deviation (mean ± SD) and compared between the two groups using the independent-samples *t*-test. Non-normally distributed variables were reported as median with first and third quartiles [M (Q1, Q3)] and analyzed using the Mann–Whitney *U*-test. Categorical data were presented as frequencies and percentages [*n* (%)] and compared using either the chi-square test or Fisher's exact test, as appropriate. For all between-group comparisons, a *P* value < 0.05 was considered statistically significant. To identify potential risk factors for postoperative AHD, univariate logistic regression was first performed with *P* < 0.05 as the selection threshold; variables meeting this criterion were then entered into a multivariate logistic regression model to determine independent risk factors, with *P* < 0.05 again considered significant. Receiver operating characteristic (ROC) curves were generated to evaluate predictive value. Flowcharts were created using GraphPad Prism software (version 9.0).

## 3 Results

### 3.1 Patient characteristics

A total of 122 patients were enrolled, comprising 100 males (81.97%) and 22 females (18.03%), with a median age of 28.00 years (IQR: 25.00–29.00). Postoperative AHD occurred in 34 patients (27.87%), who were assigned to the AHD group, while the remaining 88 patients (72.13%) formed the non-AHD group. Baseline comparisons revealed significant differences between the two groups in heart rate (HR, *P* = 0.036), white blood cell count (*P* = 0.003), neutrophil count (*P* = 0.005), lymphocyte count (*P* = 0.049), lymphocyte-to-monocyte ratio (LMR, *P* = 0.003), pan-immune-inflammation value (PIV, *P* = 0.026), systemic inflammation response index (SIRI, *P* = 0.002), total protein (TP, *P* = 0.002), albumin (*P* = 0.023), creatinine (Cr, *P* < 0.001), prothrombin time (PT, *P* = 0.036), and international normalized ratio (INR, *P* = 0.01) (Table 1).

**Table 1:** Basic characteristics of all patients included in the study.

Variables	Total (*n* = 122)	No-AHD (*n* = 88)	AHD (*n* = 34)	*P*
Gender				0.263
Male	100 (81.97)	70 (79.55)	30 (88.24)	
Female	22 (18.03)	18 (20.45)	4 (11.76)	
Smoking, *n* (%)				0.215
No	68 (55.74)	46 (52.27)	22 (64.71)	
Yes	54 (44.26)	42 (47.73)	12 (35.29)	
Drinking, *n* (%)				0.736
No	87 (71.31)	62 (70.45)	25 (73.53)	
Yes	35 (28.69)	26 (29.55)	9 (26.47)	
HP, *n* (%)				0.161
No	80 (65.57)	61 (69.32)	19 (55.88)	
Yes	42 (34.43)	27 (30.68)	15 (44.12)	
DM, *n* (%)				0.279
No	121 (99.18)	88 (100.00)	33 (97.06)	
Yes	1 (0.82)	0 (0.00)	1 (2.94)	
Marfan, *n* (%)				0.980
No	106 (86.89)	77 (87.50)	29 (85.29)	
Yes	16 (13.11)	11 (12.50)	5 (14.71)	
Age	28.00 (25.00, 29.00)	27.00 (24.00, 29.00)	28.50 (25.25, 29.00)	0.399
Height	176.92 ± 10.21	177.34 ± 10.64	175.83 ± 9.03	0.465
Weight	79.00 (66.50, 90.00)	75.75 (65.00, 90.00)	81.00 (70.00, 90.77)	0.336
BMI	25.43 (21.95, 28.16)	25.04 (21.82, 27.77)	26.80 (22.64, 28.52)	0.141
SBP	132.00 (116.00, 144.75)	132.00 (116.00, 143.00)	129.50 (115.50, 156.00)	0.670
DBP	70.95 ± 14.86	69.62 ± 14.25	74.37 ± 16.05	0.114
HP	85.65 ± 16.97	83.65 ± 17.51	90.82 ± 14.47	0.036
WBC	12.92 ± 4.01	12.25 ± 3.94	14.65 ± 3.69	0.003
Neutrophil	10.65 ± 3.95	10.04 ± 3.98	12.24 ± 3.45	0.005
Lymphocyte	1.25 (0.89, 1.73)	1.31 (0.93, 1.83)	1.02 (0.77, 1.62)	0.049
Monocyte	0.71 (0.54, 0.98)	0.71 (0.50, 0.94)	0.74 (0.60, 1.09)	0.085
RBC	4.62 (4.19, 4.93)	4.62 (4.17, 4.95)	4.60 (4.28, 4.90)	0.902
HGB	139.00 (126.00, 147.00)	139.00 (125.25, 146.00)	139.00 (126.50, 149.00)	0.579
PLT	215.50 (162.50, 254.75)	220.50 (171.50, 259.00)	201.00 (128.25, 239.25)	0.061
LMR	1.59 (1.15, 2.60)	1.77 (1.29, 2.77)	1.36 (0.91, 1.65)	0.003
PIV	1,396.78 (609.62, 2,180.93)	1,207.62 (570.90, 1,898.88)	1,784.72 (871.26, 3,052.16)	0.026
SIRI	6.70 (3.48, 10.86)	5.83 (3.22, 8.72)	9.84 (6.21, 14.24)	0.002
SII	1,750.44 (931.32, 2,865.49)	1,570.71 (886.99, 2,642.03)	2,217.30 (1,128.58, 3,692.89)	0.083
PLR	171.09 (115.90, 232.50)	157.81 (116.92, 223.15)	190.72 (107.16, 265.10)	0.422
ALT	25.75 (14.20, 46.85)	23.70 (13.47, 45.42)	29.70 (18.20, 65.77)	0.138
AST	22.70 (16.35, 39.25)	21.60 (16.00, 32.12)	31.60 (17.58, 45.45)	0.054
TP	65.08 ± 5.63	66.02 ± 5.04	62.62 ± 6.39	0.002
Albumin	40.28 ± 3.93	40.78 ± 3.59	38.99 ± 4.49	0.023
Cr	75.95 (64.00, 99.27)	70.75 (60.70, 84.50)	106.65 (90.65, 144.08)	<.001
APTT	35.65 (29.95, 39.77)	35.35 (30.25, 39.45)	37.25 (30.10, 39.77)	0.582
PT	13.15 (12.30, 14.00)	13.00 (12.28, 13.83)	13.70 (12.38, 14.47)	0.036
INR	1.04 (0.98, 1.10)	1.02 (0.97, 1.09)	1.06 (1.03, 1.17)	0.010
FIB	2.71 (2.17, 4.06)	2.73 (2.22, 4.19)	2.55 (1.85, 3.45)	0.120
TT	16.50 (15.83, 17.60)	16.40 (15.80, 17.30)	17.00 (15.93, 18.05)	0.089
LVEF	60.00 (57.00, 65.00)	60.00 (57.00, 65.00)	60.00 (56.47, 63.00)	0.815
Infusion of RBC	2.00 (0.00, 4.00)	2.00 (0.00, 4.00)	2.00 (0.00, 4.00)	0.791
Infusion of plasma	400.00 (312.50, 587.50)	400.00 (300.00, 500.00)	400.00 (362.50, 600.00)	0.355
Infusion of PLT	1.00 (0.00, 2.00)	1.00 (0.00, 4.00)	1.00 (0.00, 1.00)	0.132
CPB	206.00 (165.50, 245.00)	199.00 (156.50, 243.50)	209.00 (190.00, 247.25)	0.111
Aortic occlusion time	119.50 (84.00, 150.00)	116.00 (79.00, 155.50)	125.50 (94.00, 142.25)	0.524
DCCA	20.00 (15.00, 26.00)	20.00 (15.87, 26.00)	20.00 (13.25, 26.00)	0.637
Surgery time	425.00 (386.12, 487.50)	411.83 (373.88, 472.13)	440.00 (404.94, 518.75)	0.036

SBP, Systolic blood pressure; DBP, Diastolic blood pressure; HP, Heart rate; WBC, White blood cell; RBC, Red blood cell; HGB, Hemoglobin; PLT, Platelets; LMR, lymphocyte/monocyte; PIV, (Neutrophil × Platelet × onocyte)/lymphocyte; SIRI, (Neutrophil × monocyte)/lymphocyte; SII, (Platelet × Neutrophil)/Lymphocyte; PLR, Platelets/lymphocyte; ALT, Alanine aminotransferase; AST, Aspartate amino transferase; TP, Total protein; FIB, Fibrinogen; CPB, Cardiopulmonary bypass; DCCA, Deep hypothermia circulation arrest.

### 3.2 Univariate and multivariate logistic regression analysis

Univariate logistic regression analysis identified several factors significantly associated with postoperative AHD, including a history of hypertension (OR = 1.03, 95% CI: 1.01–1.05, *P* = 0.04), white blood cell count (OR = 1.17, 95% CI: 1.05–1.30, *P* = 0.004), neutrophil count (OR = 1.16, 95% CI: 1.04–1.29, *P* = 0.007), lymphocyte-to-monocyte ratio (LMR) (OR = 0.58, 95% CI: 0.38–0.91, *P* = 0.016), pan-immune-inflammation value (PIV) (OR = 1.01, 95% CI: 1.01–1.01, *P* = 0.018), systemic inflammation response index (SIRI) (OR = 1.10, 95% CI: 1.03–1.17, *P* = 0.003), total protein (TP) (OR = 0.89, 95% CI: 0.82–0.96, *P* = 0.004), albumin (OR = 0.89, 95% CI: 0.80–0.99, *P* = 0.027), creatinine (Cr) (OR = 1.04, 95% CI: 1.02–1.06, *P* < 0.001), and prothrombin time (PT) (OR = 1.33, 95% CI: 1.04–1.71, *P* = 0.026). Subsequent multivariate logistic regression analysis demonstrated that Cr remained the sole independent risk factor for postoperative AHD in ATAAD patients aged ≤30 years (OR = 1.04, 95% CI: 1.01–1.06, *P* = 0.002) (Table 2).

**Table 2:** Univariate and multivariate logistic regression analysis for predicting risk factors of postoperative AHD in ATAAD patients aged ≤30 years.

Variables	Univariate Logistic Regression Analysis		Multivariate Logistic Regression Analysis	
	*P*	OR (95%CI)	*P*	OR (95%CI)
Gender				
Male		1.00 (Reference)		
Female	0.269	0.52 (0.16∼1.66)		
Smoking, *n* (%)				
No		1.00 (Reference)		
Yes	0.217	0.60 (0.26∼1.35)		
Drinking, *n* (%)				
No		1.00 (Reference)		
Yes	0.737	0.86 (0.35∼2.09)		
HP, *n* (%)				
No		1.00 (Reference)		
Yes	0.164	1.78 (0.79∼4.03)		
DM, *n* (%)				
No		1.00 (Reference)		
Yes	0.991	15,354,167.63 (0.00∼Inf)		
Manfan, *n* (%)				
No		1.00 (Reference)		
Yes	0.746	1.21 (0.39∼3.77)		
Age	0.340	1.06 (0.94∼1.18)		
Height	0.462	0.99 (0.95∼1.02)		
Weight	0.212	1.01 (0.99∼1.03)		
BMI	0.063	1.07 (1.00∼1.15)		
SBP	0.156	1.01 (1.00∼1.03)		
DBP	0.116	1.02 (0.99∼1.05)		
HR	0.040	1.03 (1.01∼1.05)		
WBC	0.004	1.17 (1.05∼1.30)		
Neutrophil	0.007	1.16 (1.04∼1.29)		
Lymphocyte	0.085	0.54 (0.27∼1.09)		
Monocyte	0.051	2.87 (1.00∼8.25)		
RBC	0.657	0.86 (0.44∼1.67)		
HGB	0.803	1.00 (0.98∼1.03)		
PLT	0.069	0.99 (0.99∼1.00)		
LMR	0.016	0.58 (0.38∼0.91)		
PIV	0.018	1.01 (1.01∼1.01)		
SIRI	0.003	1.10 (1.03∼1.17)		
SII	0.082	1.00 (1.00∼1.00)		
PLR	0.401	1.00 (1.00∼1.00)		
ALT	0.139	1.01 (1.00∼1.01)		
AST	0.096	1.01 (1.00∼1.01)		
TP	0.004	0.89 (0.82∼0.96)		
Albumin	0.027	0.89 (0.80∼0.99)		
Cr	<.001	1.04 (1.02∼1.06)	0.002	1.04 (1.01∼1.06)
APTT	0.579	1.02 (0.96∼1.08)		
PT	0.026	1.33 (1.04∼1.71)		
INR	0.607	0.93 (0.72∼1.21)		
FIB	0.097	0.78 (0.59∼1.04)		
LVEF	0.883	1.00 (0.96∼1.05)		
TT	0.086	1.22 (0.97∼1.54)		
Infusion of RBC	0.929	0.99 (0.85∼1.15)		
Infusion of plasma	0.371	1.00 (1.00∼1.00)		
Infusion of PLT	0.062	0.86 (0.73∼1.01)		
CPB	0.092	1.00 (1.00∼1.01)		
Aortic occlusion time	0.636	1.00 (0.99∼1.01)		
DCCA	0.973	1.00 (0.96∼1.04)		
Surgery time	0.112	1.00 (1.00∼1.01)		

OR, odds ratio; CI, confidence interval.

### 3.3 Predictive value of ROC curve

**Figure 2:**
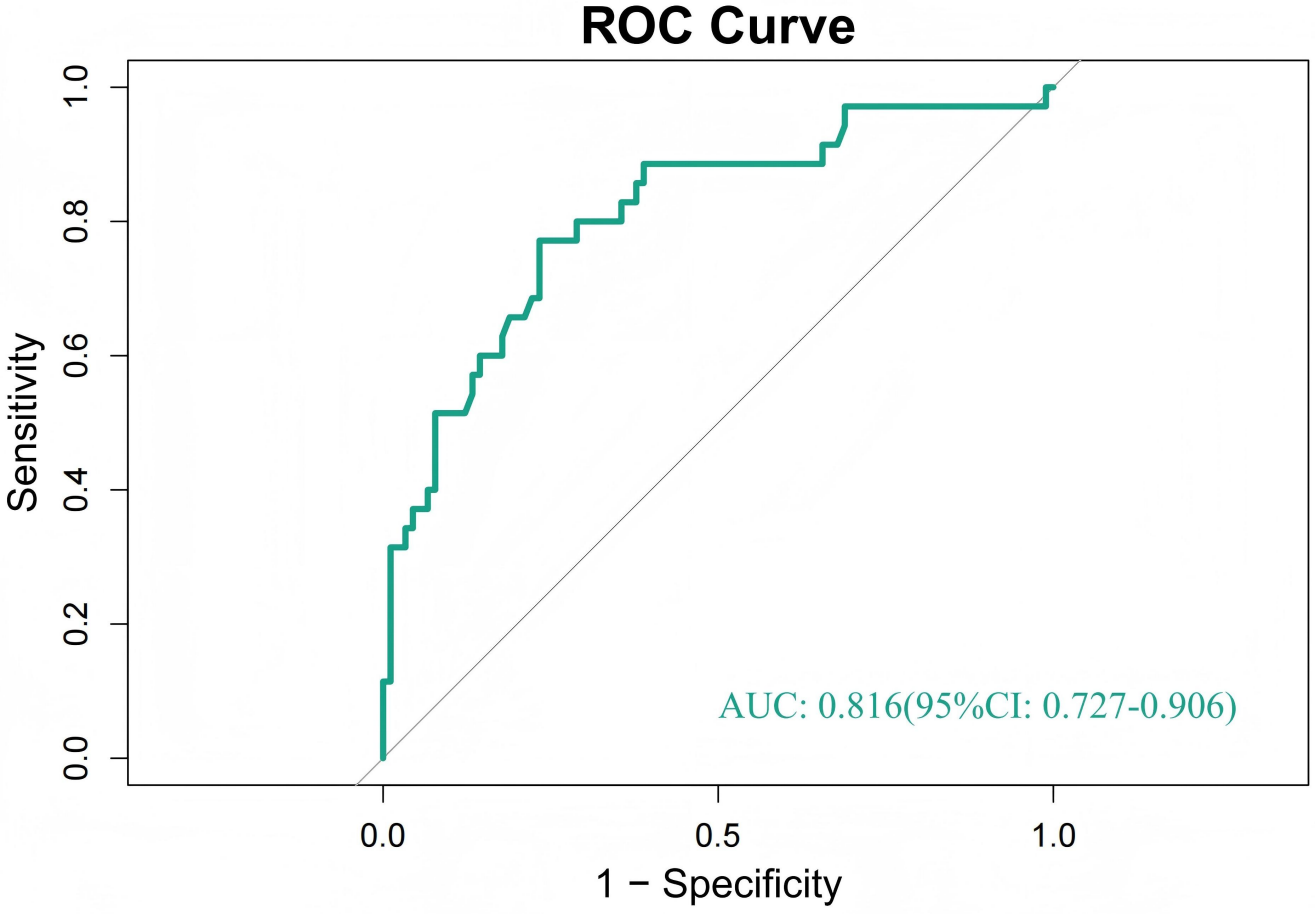
ROC curve analysis of admission serum creatinine in predicting postoperative AHD among ATAAD patients aged ≤30 years.

We constructed a receiver operating characteristic (ROC) curve to evaluate the predictive value of admission serum creatinine (Cr) for postoperative AHD. As shown in Figure 2, the area under the curve (AUC) for admission Cr levels in predicting postoperative AHD among ATAAD patients aged ≤30 years was 0.816 (sensitivity: 0.765; specificity: 0.773), indicating good predictive performance (Table 3; Figure 2).

**Table 3:** The serum creatinine level upon admission can predict postoperative AHD in patients with TAAD aged ≤30 years.

Variable	AUC	95%CI	P	Sensitivity	Specificity
Cr	0.816	0.727–0.906	＜0.0001	0.765	0.773

### 3.4 Sensitivity analysis adjusting for intraoperative variables

To assess whether admission Cr independently predicts postoperative AHD beyond intraoperative factors, we performed a sensitivity analysis by including admission Cr together with key intraoperative variables in a multivariable logistic regression model. The results showed that admission Cr remained an independent predictor of postoperative AHD (OR = 1.04, 95% CI: 1.02–1.06, *P* < .001), while none of the intraoperative variables reached statistical significance (all *P* > 0.05)(Supplementary Table S1).

## 4 Discussion

Acute type A aortic dissection is a life-threatening cardiovascular disease. The majority of patients require emergency surgery, and both in-hospital mortality and complication rates remain high. Postoperative acute hepatic dysfunction is strongly associated with in-hospital death (15, 16). Although young patients (≤30 years) generally have fewer comorbidities and better physiological reserves, acute malperfusion and the perioperative systemic inflammatory response still place them at a significantly increased risk of postoperative organ dysfunction (17, 18). Marfan syndrome was not significantly associated with postoperative AHD in the present cohort; nevertheless, its established etiological importance in young ATAAD patients warrants recognition. Previous studies have shown that Marfan-associated ATAAD is characterized by more frequent aortic root and supra-aortic vessel involvement and a greater need for extensive aortic reconstruction, although Marfan syndrome itself does not appear to increase adjusted early postoperative mortality (7, 10). This study retrospectively analyzed 122 young patients who underwent surgery for acute type A aortic dissection across multiple large tertiary hospitals. It represents the first systematic investigation of risk factors for postoperative acute hepatic dysfunction specifically in this age group. The findings confirm the potential value of admission serum creatinine levels in the early prediction of postoperative hepatic insufficiency, thereby providing both a theoretical basis and a clinical reference for perioperative management in young patients with acute type A aortic dissection.

Previous studies have identified advanced age, elevated body mass index, and increased intraoperative red blood cell transfusion volume as independent risk factors for postoperative acute hepatic dysfunction (AHD) in patients with acute type A aortic dissection (ATAAD) (13, 16, 19). However, for younger patients—particularly those aged ≤30 years—advanced age is not a relevant risk factor. In this study, the incidence of postoperative AHD among ATAAD patients aged ≤30 years was 27.9%, which is slightly higher than that reported in all-age populations. This finding suggests that although younger patients lack traditional risk factors such as advanced age, they may paradoxically face a higher risk of postoperative AHD. Therefore, identifying the specific independent risk factors for postoperative AHD in young ATAAD patients (≤30 years) is essential for preoperative risk stratification, the development of targeted intraoperative protective strategies, and early postoperative intervention.

This study reveals that among patients aged ≤30 years with acute type A aortic dissection (ATAAD), a higher admission serum creatinine level is associated with an increased risk of postoperative acute hepatic dysfunction (AHD). The potential mechanisms underlying this association include the following. First, despite young patients generally having a favorable baseline physical status, ATAAD itself constitutes a severe and sudden critical illness. An elevated admission creatinine level already indicates organ impairment and diminished systemic tolerance and compensatory capacity (6, 20). The subsequent additive insults of surgical trauma and cardiopulmonary bypass may readily precipitate a cascade of liver and kidney failure, thereby elevating the risk of AHD (19, 21). Second, elevated creatinine reflects renal dysfunction, leading to the accumulation of metabolic wastes such as urea and organic acids. These toxins are predominantly transported to the liver for catabolism, overwhelming its compensatory reserve. The additional burden imposed by surgical trauma further compromises liver function and triggers AHD (22–24). Third, the presence of ATAAD often results in multiorgan malperfusion due to false lumen flow. Following emergency surgical repair and revascularization, the previously ischemic liver undergoes rapid reperfusion, which induces ischemia–reperfusion injury. This process aggravates hypoxic necrosis of hepatocytes, ultimately leading to hepatic dysfunction or failure (25, 26).

In recent years, hypertension and aortic dissection have shown a trend toward affecting younger populations. However, studies focusing on postoperative risks in ATAAD patients aged≤30 years remain very limited. Although this group generally has a better baseline physical status, they exhibit unique pathophysiological responses and demand higher long-term quality of life. This study found that admission serum creatinine (Cr) is an independent risk factor for postoperative acute hepatic dysfunction (AHD) in ATAAD patients aged ≤30 years, and that the incidence of AHD in this group is higher than that reported in all-age populations from previous studies. Therefore, greater attention should be paid to monitoring postoperative complications in young ATAAD patients, including active perioperative assessment of liver function changes and early intervention for potential severe complications. Furthermore, individualized diagnosis and treatment for critically ill patients may contribute to improved outcomes.

## 5 Limitations

This study has several limitations. First, the retrospective design may introduce selection bias and residual confounding factors that cannot be fully excluded. Second, although this is a multi-center study, the sample size is relatively limited-especially given that young ATAAD patients constitute a rare population. Therefore, larger prospective studies are needed to confirm our findings. Third, this study did not perform a stratified analysis according to the severity of AHD. Fourth, the multivariable logistic regression analysis included 10 candidate variables despite only 34 AHD events, which may increase the risk of model overfitting. Given the exploratory nature of this study—as the first investigation of postoperative AHD risk factors specifically in ATAAD patients aged ≤30 years—we retained the full model to comprehensively assess potential predictors. Nevertheless, our findings should be interpreted with caution and require external validation in larger, prospective cohorts.

## 6 Conclusion

This study demonstrates that, in patients aged ≤30 years with ATAAD, admission serum creatinine serves as an independent predictor of postoperative AHD. Clinically, careful assessment of baseline renal function upon admission and close monitoring of perioperative hepatic function changes are warranted. The integration of early strategies to mitigate surgical trauma and cardiopulmonary bypass-induced injury may reduce the risk of AHD, ultimately enhancing both short-term recovery and long-term quality of life in this young patient population.

## Data Availability

The original contributions presented in the study are included in the article/Supplementary Material, further inquiries can be directed to the corresponding author.

## References

[1] Teurneau-Hermansson K Ede J Larsson M Linton G Von Rosen D Sjögren J. Mortality after non-surgically treated acute type A aortic dissection is higher than previously reported. Eur J Cardiothorac Surg. (2024) 65(2):ezae039. 10.1093/ejcts/ezae039 38310329 PMC10871943

[2] Nakai C Izumi S Haraguchi T Henmi S Nakayama S Mikami T. Impact of time from symptom onset to operation on outcome of repair of acute type A aortic dissection with malperfusion. J Thorac Cardiovasc Surg. (2023) 165(3):984–991.e1. 10.1016/j.jtcvs.2021.03.102 33941373

[3] Hofmann Bowman MA Eagle KA. Challenges and opportunities in aortic dissection: the journey to personalized medicine. Circulation. (2024) 150(15):1155–7. 10.1161/CIRCULATIONAHA.124.071088 39374333

[4] Hameed I Cifu AS Vallabhajosyula P. Management of thoracic aortic dissection. JAMA. (2023) 329(9):756–7. 10.1001/jama.2023.0265 36795378

[5] M. Belyaev A. A comprehensive review of acute Type A aortic dissection: epidemiology, classification, management strategies, mortality risk assessment, and ethical considerations for patients who refuse blood transfusions. Rev Cardiovasc Med. (2025) 26(10):44307. 10.31083/RCM44307 41209138 PMC12593736

[6] Xie Q Zhong Y Xu Q Wang J Ge Y Li C. Early and long-term outcomes of young adult patients ≤30 years old with acute type A aortic dissection. Eur J Cardiothorac Surg. (2023) 64(6):ezad330. 10.1093/ejcts/ezad330 37758246

[7] Luehr M Yildiz M Ma WG Heck R Polycarpou A Jassar A. Acute type A aortic dissection in adolescents and young adults under 30 years of age: demographics, aetiology and postoperative outcomes of 139 cases. Eur J Cardiothorac Surg. (2023) 63(5):ezad112. 10.1093/ejcts/ezad112 36951534

[8] Lin XF Gao HQ Wu QS Xie YL Chen LW Xie LF. Demographics and outcomes of acute type A aortic dissection in young adults in southeastern China: impact of syndromic heritable thoracic aortic disease. Ann Med. (2025) 57(1):2457530. 10.1080/07853890.2025.2457530 39873639 PMC11776063

[9] Nucera M Heinisch PP Langhammer B Jungi S Mihalj M Schober P. The impact of sex and gender on aortic events in patients with Marfan syndrome. Eur J Cardiothorac Surg. (2022) 62(5):ezac305. 10.1093/ejcts/ezac305 35543473

[10] Farag M Büsch C Rylski B Pöling J Dohle DS Sarvanakis K. early outcomes of patients with Marfan syndrome and acute aortic type A dissection. J Thorac Cardiovasc Surg. (2023) 166(1):25–34.e8. 10.1016/j.jtcvs.2021.07.024 34446289

[11] Zhou WJ Wu HY Wang JX Liu SD Xu YP Song B. Acute aortic dissection-induced acute respiratory distress syndrome: pathogenesis and clinical implications. Front Cardiovasc Med. (2025) 12:1654456. 10.3389/fcvm.2025.1654456 41357087 PMC12679279

[12] Yang J Ji D Zhu YQ Ren Y Zhang X Dai HY. Hemoperfusion with HA380 in acute type A aortic dissection patients undergoing aortic arch operation (HPAO): a randomized, controlled, double-blind clinical trial. Trials. (2020) 21(1):954. 10.1186/s13063-020-04858-2 33228727 PMC7684885

[13] Huo Y Zhang H Li B Zhang K Li B Guo SH. Risk factors for postoperative mortality in patients with acute stanford Type A aortic dissection. Int J Gen Med. (2021) 14:7007–15. 10.2147/IJGM.S330325 34707392 PMC8544269

[14] Lai JC Covinsky KE Dodge JL Boscardin WJ Segev DL Roberts JP. Development of a novel frailty index to predict mortality in patients with end-stage liver disease. Hepatology. (2017) 66(2):564–74. 10.1002/hep.29219 28422306 PMC5519430

[15] Xu Y Liu LZ Lu HQ Yang XQ Guo SK Tang YJ. Risk factors for postoperative hepatic dysfunction in overweight patients with acute type A aortic dissection. BMC Surg. (2024) 24(1):305. 10.1186/s12893-024-02621-x 39396012 PMC11470697

[16] Sheng W Qiao H Wang Z Niu Z Lv X. Analysis of prognosis and risk factors for postoperative hepatic dysfunction in patients with acute Type A aortic dissection. Anatol J Cardiol. (2023) 27(4):197–204. 10.14744/AnatolJCardiol.2022.2644 36995052 PMC10098375

[17] Chandiramani A Al-Tawil M Elleithy A Kakar S Rajasekar T Panda A. Organ-specific malperfusion in acute type A aortic dissection: epidemiological meta-analysis of incidence rates. BJS Open. (2024) 9(1):zrae146. 10.1093/bjsopen/zrae146 39792052 PMC11720173

[18] Li J Wang X Zhu K Jin X Zhang H. Perioperative dynamic changes of systemic inflammatory response, gut injury, and hypoxemia in patients with acute type-A aortic dissection: an observational case-control study. J Thorac Dis. (2025) 17(2):1054–63. 10.21037/jtd-2025-141 40083518 PMC11898389

[19] Han X Wang W Gu T Shi E. Development and validation of a predictive model for postoperative hepatic dysfunction in Stanford type A aortic dissection. Sci Rep. (2025) 15(1):22126. 10.1038/s41598-025-06024-7 40596101 PMC12215675

[20] Ahmad D Sá MP Diaz Castrillon CE Thoma F Wang Y Kaczorowski D. Trends in kidney function and chronic kidney disease after surgery for acute Type A aortic dissection. Ann Vasc Surg. (2025) 112:139–47. 10.1016/j.avsg.2024.11.098 39681215

[21] Salameh A Dhein S. Strategies for pharmacological organoprotection during extracorporeal circulation targeting ischemia-reperfusion injury. Front Pharmacol. (2015) 6:296. 10.3389/fphar.2015.00296 26733868 PMC4686733

[22] Baker PR 2nd Li AS Griffin BR Gil HW Orlicky DJ Fox BM. Disruption in glutathione metabolism and altered energy production in the liver and kidney after ischemic acute kidney injury in mice. Sci Rep. (2024) 14(1):13862. 10.1038/s41598-024-64586-4 38879688 PMC11180093

[23] Lee SA Cozzi M Bush EL Rabb H. Distant organ dysfunction in acute kidney injury: a review. Am J Kidney Dis. (2018) 72(6):846–56. 10.1053/j.ajkd.2018.03.028 29866457 PMC6252108

[24] Zhou W Wang G Liu Y Tao Y Du Z Tang Y. Outcomes and risk factors of postoperative hepatic dysfunction in patients undergoing acute type A aortic dissection surgery. J Thorac Dis. (2019) 11(8):3225–33. 10.21037/jtd.2019.08.72 31559024 PMC6753406

[25] Lin X Xie L Jiang D Wu Q He J Chen L. Hepatic dysfunction and adverse outcomes after total arch repair of acute type a aortic dissection: application of the MELD-XI score. BMC Cardiovasc Disord. (2022) 22(1):491. 10.1186/s12872-022-02934-w 36401191 PMC9673427

[26] Kobayashi N Takano M Shirakabe A Hata N Kawamata H Mizuno K. Intravascular ultrasound-guided endovascular stenting for celiac artery complicated with hepatic hypoperfusion after acute type B aortic dissection. J Am Coll Cardiol. (2012) 59(17):1568. 10.1016/j.jacc.2011.07.063 22516449

