# Supplementary material for: Admission creatinine level predicts postoperative acute hepatic dysfunction in patients aged ≤30 years with acute type A aortic dissection: a multi-center retrospective study
Source: Front Cardiovasc Med. 2026 Aug 17;13:1898082. doi: 10.3389/fcvm.2026.1898082 (PMC13527447; doi:10.3389/fcvm.2026.1898082)
Supplement: Supplementary file 1 [file Table1.docx]

Supplementary Table 1 Sensitivity analysis adjusting for intraoperative variables

| Variables | Univariate Logistic Regression Analysis | | Multivariate Logistic Regression Analysis | |
| --- | --- | --- | --- | --- |
|  | *P* | OR (95%CI) | *P* | OR (95%CI) |
| HR | 0.040 | 1.03 (1.01 ~ 1.05) |  |  |
| Cr | <.001 | 1.04 (1.02 ~ 1.06) | <.001 | 1.04 (1.02 ~ 1.06) |
| Infusion of RBC | 0.929 | 0.99 (0.85 ~ 1.15) |  |  |
| Infusion of plasma | 0.371 | 1.00 (1.00 ~ 1.00) |  |  |
| Infusion of PLT | 0.062 | 0.86 (0.73 ~ 1.01) |  |  |
| CPB time | 0.092 | 1.00 (1.00 ~ 1.01) |  |  |
| Aortic occlusion time | 0.636 | 1.00 (0.99 ~ 1.01) |  |  |
| DCCA time | 0.973 | 1.00 (0.96 ~ 1.04) |  |  |
| OR: Odds Ratio, CI: Confidence Interval | | | | |
